# Mobilization of innate and adaptive antitumor immune responses by the RNP-targeting antibody ATRC-101

**DOI:** 10.1073/pnas.2123483119

**Published:** 2022-05-04

**Authors:** Alexander Scholz, Jeff DeFalco, Yvonne Leung, Iraz T. Aydin, Cathrin J. Czupalla, Wei Cao, Daniel Santos, Nikhil Vad, Shaun M. Lippow, Gilson Baia, Michael Harbell, Judevin Sapugay, Danhui Zhang, Dai-Chen Wu, Erin Wechsler, Anne Z. Ye, Jenny W. Wu, Xiao Peng, John Vivian, Hargita Kaplan, Rodney Collins, Ngan Nguyen, Mark Whidden, Dongkyoon Kim, Carl Millward, Jonathan Benjamin, Norman M. Greenberg, Tito A. Serafini, Daniel E. Emerling, Lawrence Steinman, William H. Robinson, Amy Manning-Bog

**Affiliations:** ^a^Atreca, Inc, San Carlos, CA 94070;; ^b^Department of Neurology and Neurological Sciences and Pediatrics, Stanford University, Stanford, CA 94305;; ^c^Division of Immunology and Rheumatology, Stanford University School of Medicine, Stanford, CA 94305

**Keywords:** tumor immunology, ribonucleoprotein, personalized tumor therapy

## Abstract

Immunotherapy approaches focusing on T cells have provided breakthroughs in treating solid tumors. However, there remains an opportunity to drive anticancer immune responses via other cell types, particularly myeloid cells. ATRC-101 was identified via a target-agnostic process evaluating antibodies produced by the plasmablast population of B cells in a patient with non-small cell lung cancer experiencing an antitumor immune response during treatment with checkpoint inhibitor therapy. Here, we describe the target, antitumor activity in preclinical models, and data supporting a mechanism of action of ATRC-101. Immunohistochemistry studies demonstrated tumor-selective binding of ATRC-101 to multiple nonautologous tumor tissues. In biochemical analyses, ATRC-101 appears to target an extracellular, tumor-specific ribonucleoprotein (RNP) complex. In syngeneic murine models, ATRC-101 demonstrated robust antitumor activity and evidence of immune memory following rechallenge of cured mice with fresh tumor cells. ATRC-101 increased the relative abundance of conventional dendritic cell (cDC) type 1 cells in the blood within 24 h of dosing, increased CD8^+^ T cells and natural killer cells in blood and tumor over time, decreased cDC type 2 cells in the blood, and decreased monocytic myeloid-derived suppressor cells in the tumor. Cellular stress, including that induced by chemotherapy, increased the amount of ATRC-101 target in tumor cells, and ATRC-101 combined with doxorubicin enhanced efficacy compared with either agent alone. Taken together, these data demonstrate that ATRC-101 drives tumor destruction in preclinical models by targeting a tumor-specific RNP complex leading to activation of innate and adaptive immune responses.

## Significance

A target-agnostic approach that harnesses the human antitumor immune response to find potential anticancer lead antibodies and their targets was used to generate ATRC-101, an engineered version of a tumor-targeting antibody identified from a patient with non-small cell lung cancer experiencing an ongoing antitumor immune response. ATRC-101 is an antibody that targets an extracellular, tumor-specific ribonucleoprotein complex. Here, we describe the extracellular binding of this complex and antitumor activity of ATRC-101 in murine models. Preclinical data suggest a mechanism of action in which ATRC-101 activates myeloid cells of the innate immune system, leading to an adaptive immune response that yields its antitumor activity. These data have led to an ongoing phase 1 trial in patients with advanced solid tumors.

Advances in immunotherapy have provided a breakthrough in cancer treatment by introducing options beyond the previous mainstay of cytotoxic chemotherapy and targeted molecular therapies (1). Antibodies that target immune checkpoint proteins, such as cytotoxic T lymphocyte antigen-4 (CTLA-4), programmed death-1 (PD-1), and programmed death-ligand 1 (PD-L1), have been studied and approved for the treatment of various cancers (1, 2). While immune checkpoint inhibitors (ICIs) have shown promising results in various cancer types, not all patients benefit from treatment with some developing immune-related adverse events and the acquisition of resistance (3–9).

The benefits of T-cell directed immunotherapy for a subset of patients with cancer has invigorated efforts to exploit the antitumor capabilities of other immune cells. Myeloid cells play a crucial role in the immune system’s ability to recognize self from nonself (10). One mechanism by which this effect is mediated is the recognition of nucleic acids in the extracellular environment by endosomal toll-like receptors (TLRs), which can activate dendritic cells (DC), leading to production and release of cytokines, such as type 1 interferon (10–12). Studies of TLR stimulation have shown antitumor effects through various mechanisms, including reducing myeloid-derived suppressor cells (MDSCs), activating natural killer (NK) cells, and inducing cytotoxic T cells (13–16). B cells also play a key role in the immune response, including maintaining innate immunity and producing proinflammatory and regulatory cytokines (17). Engaging multiple immune-cell types may represent a step forward for immuno-oncology therapies.

During an immune response, antigen-activated B cells differentiate into plasmablasts and memory B cells (18). More than 75% of plasmablasts circulating in the peripheral blood express antibodies that are specific to antigens of the ongoing immune response (18); given their relatively short persistence in the bloodstream, blood plasmablasts can therefore provide insights into the antigens being actively processed by the immune system and the antibodies being generated by affinity maturation at a particular point in time (i.e., the immune repertoire) (18, 19). In addition, higher plasmablast levels have been reported in the blood of patients with nonprogressive metastatic cancer compared with healthy individuals (19). The analysis of plasmablasts collected from patients with ongoing antitumor responses enables the generation of antibody-based candidate cancer therapeutics and tumor targets through the interrogation of the human immune system to find potential targets. The antibodies produced by patients’ plasmablasts are screened to identify those that bind selectively to tumor tissues. ATRC-101 is a therapeutic candidate identified using this approach. Here we describe the identification and preclinical characterization of ATRC-101, which is currently in a phase 1b clinical trial in patients with advanced solid tumors.

## Results

### Tumor-Selective Binding of ATRC-101.

**Fig. 1.**
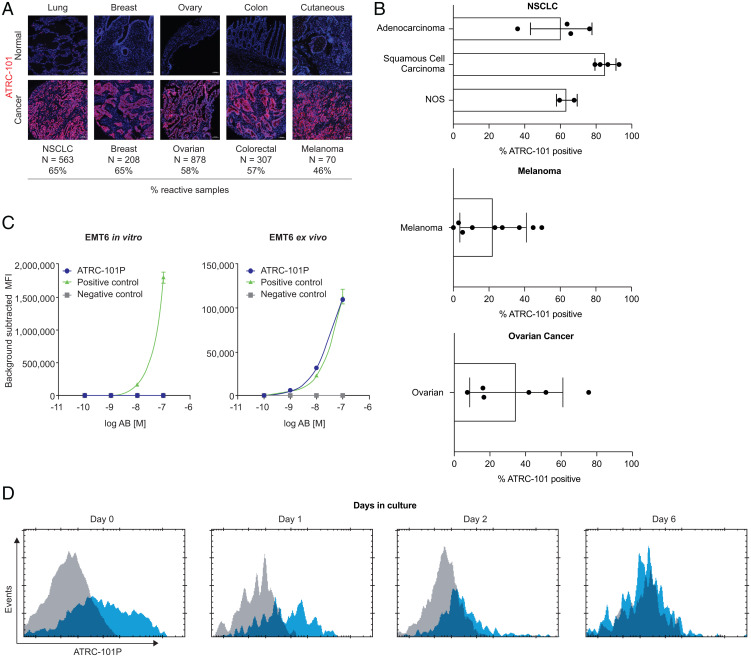
ATRC-101 reactivity is tumor-specific across multiple human cancer types. (*A*) Immunofluorescence staining of tumor versus tumor-adjacent tissue or benign tissue revealed tumor-selective binding. Reactivity was defined as “moderate or greater” on a four-point scale, with at least 40% of malignant cells estimated to be positive. Samples were largely from treatment-naïve patients. The samples on the *Top Row* from left to right are from normal adjacent lung tissue, benign breast fibroadenoma, normal adjacent ovary tissue, normal adjacent colon tissue, and benign nevus. The samples on the *Bottom Row* from left to right are from lung adenocarcinoma, breast invasive ductal carcinoma, ovary adenocarcinoma, colon adenocarcinoma, and cutaneous melanoma. Bar = 100 µm. N indicates number of individual donors analyzed. (*B*) ATRC-101 binding to the surface of primary dissociated human NSCLC, melanoma, and ovarian tumor cells as detected by flow cytometry. (*C*) Flow cytometry data for EMT6 tumor cells collected ex vivo and cells grown in vitro. (*D*) Cells from harvested ex vivo EMT6 tumors lose the target over time. Gray-filled histogram = isotype, blue-filled histogram = ATRC-101P. AB, antibody; [M], molarity; MFI, mean fluorescence intensity; NOS, not otherwise specified.

ATRC-101 was developed from an antibody (ATRC-101P) that was isolated from a patient with non-small cell lung cancer (NSCLC) who demonstrated stable disease while treated with a checkpoint inhibitor. Human immunofluorescence (IF) data using ATRC-101 revealed reactivity in a majority of samples from different (i.e., nonautologous) patients with lung, breast, ovarian, colorectal, and acral melanoma cancers (Fig. 1*A*), and a smaller percentage of samples were reactive in some other solid tumor types. A positive signal was detected in malignant cells in tumors but not in stromal cells, with no reactivity apparent in normal adjacent tissues. Thus, the ATRC-101 target appears to be tumor-specific.

Flow cytometry experiments demonstrated the binding of ATRC-101 to the surface of primary human NSCLC, ovarian, and melanoma samples collected ex vivo (Fig. 1*B*). Ten of 10 human NSCLC samples showed ATRC-101 surface binding, with 35 to 90% of EpCAM+E-cadherin+ tumor cells showing positive staining. In 5 of 9 melanoma specimens, ATRC-101 binding was detectable in more than 20% of total CD45^–^ tumor cells. Similarly, ATRC-101 surface binding was detected in 6 of 6 ovarian cancer samples with a range of tumor positivity from 7 to 75%. Flow cytometry data using ATRC-101P revealed that the target of the antibody is present on the surface of cells from mouse-derived EMT6 (Fig. 1*C*) and CT26 tumors. ATRC-101 binding to EMT6 tumor cells collected ex vivo, but not to cells grown in vitro, may indicate dependence on the tumor microenvironment for surface target localization and/or expression (Fig. 1*C*), consistent with data showing that harvested tumor cells lose surface target over time in culture (Fig. 1*D*). These data, when taken together, indicate that ATRC-101 recognizes a tumor-specific target that is present on the surface of malignant cells when in the natural tumor microenvironment.

### Initial Target Complex Characterization.

**Fig. 2.**
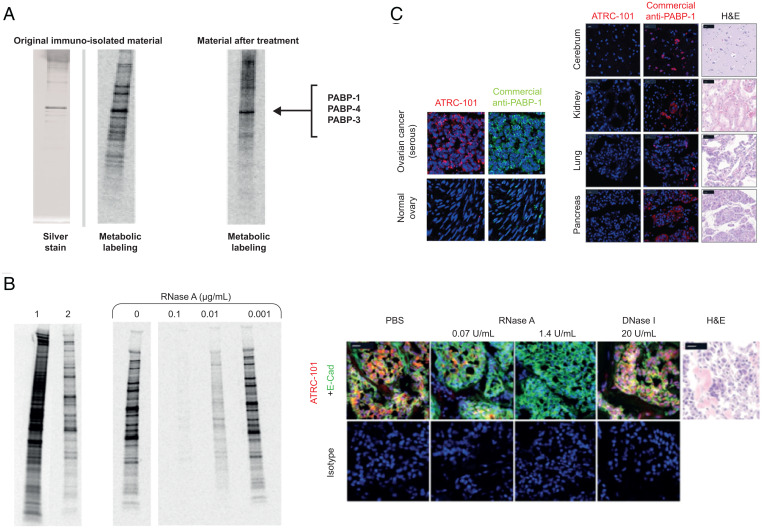
The target of ATRC-101 appears to be an RNP complex that includes PABP-1. (*A*) Cross-linking and mass-spectrometry experiments demonstrated that PABPs are abundant. (*B*) RNase A attenuated recognition of the RNA-protein complex by ATRC-101P in human tumor tissue in biochemical and histochemical experiments; 1 = whole cell lysate; 2 = ATRC-101P immunoisolate; remaining lanes represent immunoisolates in samples treated with RNase A; Bar = 20 µm. (*C*) Staining with ATRC-101 was tumor-specific versus a commercially available monoclonal PABP-1 antibody that was reactive in tumor tissue but, unlike ATRC-101, demonstrated signal in normal tissues; Bar = 20 µm. E-cad, E-cadherin; H&E, hematoxylin and eosin.

Immunoisolation of the target of ATRC-101 from A549 cells under stringent conditions with reversible cross-linking and ribonuclease (RNase) treatment (Fig. 2 *A*, *Left*) followed by mass spectroscopy revealed a complex of proteins, including many known to bind RNA, the most prominent of which being the polyadenylate binding proteins (PABP-1, -3, and -4). Other proteins present in this isolated complex included PRPF8, SNRNP200, DHX9, SF3B3, DHX30, UPF1, DHX36, MOV10, ILF3, IGF2BP3, IGF2BP1, HNRNPK, EIF4A3, ACTB, HNRNPA2B1, HNRNPC, SRSF1. The presence of many RNA-binding proteins suggests that ATRC-101 targets an RNA-binding protein complex. Immunoisolation and histochemical experiments revealed that RNase treatment attenuated, in a concentration-dependent manner, the recognition of the larger RNA-protein complex by ATRC-101, indicating that RNA is required for the antibody to associate with the full protein complex (Fig. 2 *B*, *Left*). Immunofluorescence data obtained from human breast tumor tissues also demonstrated that RNase treatment prior to ATRC-101 staining diminished immunoreactivity compared to treatment with deoxyribonuclease (DNase) or with phosphate-buffered saline (PBS) (Fig. 2 *B*, *Right*).

To further investigate whether the target of ATRC-101 is selectively expressed in tumor cells, staining patterns of ATRC-101 were compared with those of a commercial monoclonal antibody recognizing the major target complex protein, PABP-1. The commercial antibody exhibited widespread staining in normal and cancerous human samples, as would be expected based on the known ubiquitous expression of PABP-1 as an RNA-binding protein that binds polyadenylate sequences, whereas ATRC-101 reactivity was limited to the tumor with no detectable signal in normal tissues (Fig. 2*C*).

### Antitumor Activity of ATRC-101 Observed in EMT6 (breast) and CT26 (colorectal) Tumor Models.

**Fig. 3.**
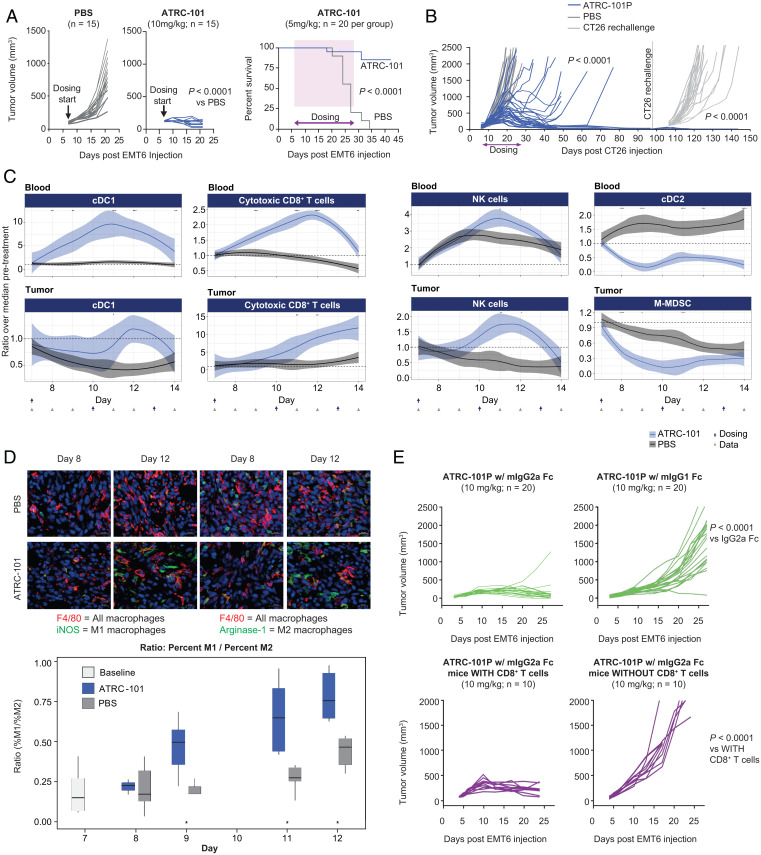
Activity of ATRC-101 in EMT6 (breast) and CT26 (colorectal) tumor models. (*A*) Treatment with ATRC-101 limited tumor growth and prolonged survival in the EMT6 tumor syngeneic mouse model. (*B*) Dosing with ATRC-101P (blue) versus PBS (gray) in the CT26 model led to no detectable tumor in 21 of 40 mice. Immune memory that prevented tumor growth was noted after rechallenge with CT26 tumor cells in all 21 of the cured mice (blue), but not in a naïve, age-matched cohort (light gray). (*C*) ATRC-101 leads to rapid changes in immune-cell profiles in the blood and tumor. Changes in immune cell numbers in EMT6 mice were quantified by multicolor flow cytometry. ATRC-101 induced almost-immediate statistically significant increases over PBS vehicle in cDC1s (CD11c^+^MHCII^+^CD103^+^CD11b^–^F4/80^–^) and cytotoxic T cells (CD3^+^CD8^+^) in the blood and subsequent statistically significant increases in tumors. Decreased cDC2s (CD11c^+^MHCII^+^CD103^–^CD11b^+^F4/80^–^) in blood at 24 h, decreased M-MDSCs (CD11b^+^Ly6Chigh) in tumors at 24 h, and increased NK cell numbers in blood and tumors at 96 h are also shown. The curves represent locally estimated scatterplot smoothing of the data by local polynomial regression fitting with shared 95% CI. *P < 0.05, **P < 0.01, ***P < 0.001, and ****P < 0.0001. (*D*) ATRC-101 induced a shift toward M1 macrophages from Day 8 to Day 12. Immunohistochemistry images (*Top*) and changes in M1/M2 ratios (*Bottom*; ratios of the percentage of nucleated cells that are F4/80^+^iNOS^+^ relative to the percentage of nucleated cells that are F4/80^+^Arginase-1^+^) in EMT6 tumors. Bar = 20 µm. (*E*) ATRC-101 activity requires both innate and adaptive immune responses. The activity of ATRC-101P is abolished when the IgG is swapped from mIgG2a (*Top*). Activity of ATRC-101P is also abolished when the experiment is performed in mice that underwent antibody-mediated CD8^+^ T-cell depletion (*Bottom*). Two-way ANOVA with *P < 0.05. cDC1, conventional type 1 dendritic cell; cDC2, conventional type 2 dendritic cell; IgG, immunoglobulin G; iNOS, inducible nitric oxide synthase.

To evaluate the antitumor activity of ATRC-101, in vivo experiments were performed in the syngeneic EMT6 (breast) and CT26 (colorectal) tumor models. Treatment with ATRC-101 led to a striking decrease in EMT6 in vivo tumor growth versus PBS vehicle (*P* < 0.0001; Fig. 3 *A*, *Left*), as well as a statistically significant survival benefit (*P* < 0.0001; Fig. 3 *A*, *Right*). Antitumor activity was also observed in the CT26 model. Following a washout period of greater than 20 half-lives, cured mice as well as naïve, age-matched controls were rechallenged with CT26 cells and no additional dosing. Although control mice revealed rapid CT26 proliferation, no significant tumor growth was observed in animals originally treated with ATRC-101P indicating immune memory in the treated mice (*P* < 0.0001; Fig. 3*B*).

Time-course experiments focusing on the antitumor immune response were performed in EMT6 mice to gain insight into the mechanism of ATRC-101. These experiments showed increases in the blood of conventional dendritic cell type 1 (cDC1) and cytotoxic CD8^+^ T cells (by flow cytometry; Fig. 3 *C*, *Left, Top Two*), followed by corresponding increases of the same cell types in the tumor (by flow cytometry; Fig. 3 *C*, *Left, Bottom Two*). NK cell levels increased with ATRC-101 at approximately the same timing as increases in CD8^+^ T cells (Fig. 3 *C*, *Third Column*). Among the earliest changes to occur (observed 24 h after dosing) were a decrease in the blood of conventional dendritic cell type 2 (cDC2) cells (contrasting with increases in cDC1 cells above) and a decrease in monocytic myeloid-derived suppressor cells (M-MDSCs) in the tumor (Fig. 3 *C*, *Fourth Column*). Treatment with ATRC-101 also induced a shift in tumor-associated macrophages toward the M1 (inflammatory antitumorigenic) phenotype (Fig. 3*D*).

To understand more about the role of the innate and adaptive immune systems in the activity of ATRC-101, the interaction of the Fc receptor on myeloid cells and the requirement for CD8^+^ T cells were evaluated in EMT6 mice. ATRC-101P antitumor activity is abolished when the mouse IgG2a (mIgG2a) Fc domain, which interacts with Fc receptors on mouse myeloid cells, is replaced by the mouse IgG1 (mIgG1) Fc domain, which does not interact with such cells (Fig. 3 *E*, *Top Two*); this indicates that myeloid cells have a key role in the activity of ATRC-101. A requirement for CD8^+^ T cells in the adaptive immune response was demonstrated by antibody-mediated depletion of CD8^+^ T cells in Balb/c mice (Fig. 3 *E*, *Bottom Two*).

### Induction of Myeloid Cell Activity Driven by the Target of ATRC-101.

**Fig. 4.**
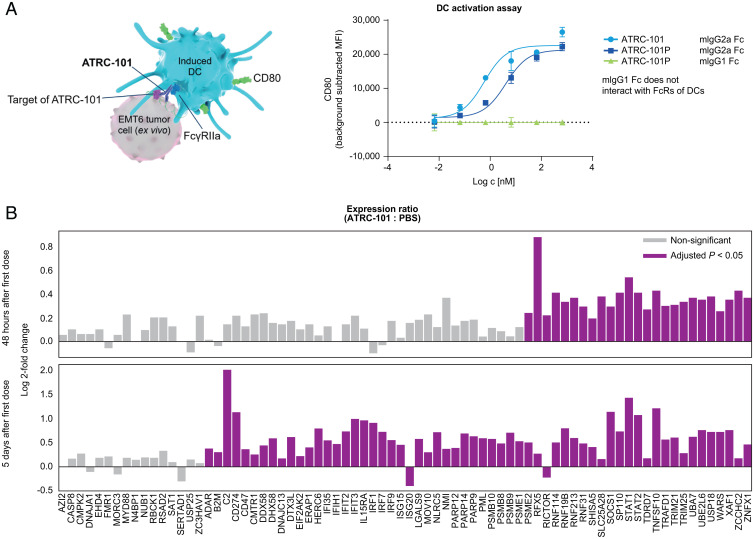
In vitro assay examining markers of DC activation (*A*) The target of ATRC-101 activated DCs in an ATRC-101–dependent manner. ATRC-101 opsonized tumor cells induced an activated phenotype of bone marrow-derived DC in a dose-dependent manner. (*B*) Coculture of EMT6 ex vivo and bone marrow-derived DCs in the presence of ATRC-101 or ATRC-101P led to a dose-dependent increase of the costimulatory surface molecule CD80. In contrast, ATRC-101P on an mIgG1 Fc background, which does not interact with myeloid Fc-gamma receptors, failed to induce an activated DC phenotype. (*B*) ATRC-101 induced significant increases in ISG expression in tumors. FcR, Fc receptor; Ig, immunoglobulin; ISG, interferon-stimulated gene; MFI, mean fluorescence intensity.

To examine the effect of ATRC-101 on DC activation in vitro, we assessed the induction of activation markers on the surface of primary murine bone marrow-derived dendritic cells (BMDCs) after exposure to ATRC-101–opsonized EMT6 ex vivo cells (Fig. 4*A*). As shown in Fig. 4*A*, coculture of EMT6 ex vivo cells and BMDCs in the presence of ATRC-101 or ATRC-101P leads to a dose-dependent increase of the costimulatory surface molecule CD80. In contrast, ATRC-101P on a mIgG1 Fc background, which does not interact with myeloid Fc-gamma receptors, failed to induce an activated DC phenotype (Fig. 4*A*). Activated myeloid cells, including a subset of DCs, are potent inducers of a T-cell antitumor response. To assess this effect in vivo, we examined the interferon response through mRNA seq analysis of primary tumor samples from tumor-bearing mice treated with ATRC-101 (Fig. 4*B*). Compared to control, ATRC-101 led to a significant up-regulation of multiple interferon-stimulated genes (ISG) at 48 h after the first dose, which was further amplified at 5 d post-first dose (Fig. 4*B*).

### Enhanced Activity of ATRC-101 in Combination with Chemotherapy.

**Fig. 5.**
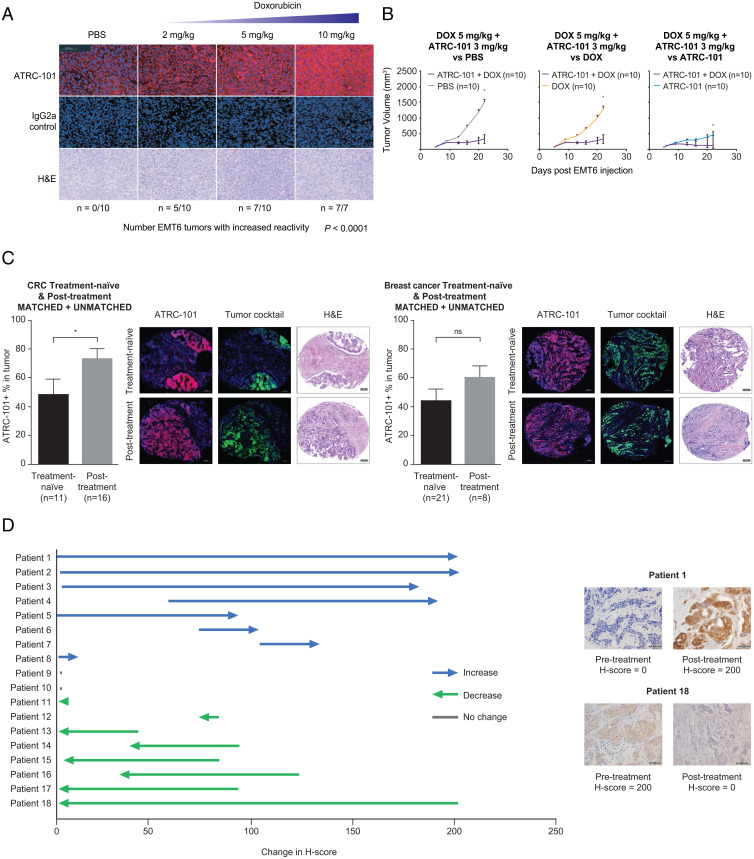
Impact of therapeutics on ATRC-101 efficacy in mice and immunoreactivity in human carcinomas. (*A*) Doxorubicin increased signal for the target of ATRC-101 at dose levels that have limited effect on tumor growth; Bar = 100 µm. (*B*) Combination therapy showed significantly enhanced antitumor activity versus PBS, doxorubicin alone, and ATRC-101 alone in EMT6 mouse model. (*C*) CRC samples from treated patients have an increased percentage of mATRC-101–immunoreactive cells versus samples from treatment-naïve patients (*Left*), and breast cancer samples from treated patients have an increased percentage of mATRC-101–immunoreactive cells versus samples from treatment-naïve patients (*Right*). Bar = 100 µm. (*D*) Standard-of-care neoadjuvant treatment changes in immunoreactivity of ATRC-101 in pre- versus posttreatment human FFPE breast cancer tissue samples. Bar = 50 µm *P < 0.05. CRC, colorectal cancer; DOX, doxorubicin; FFPE, formalin-fixed paraffin-embedded; H&E, hematoxylin and eosin; Ig, immunoglobulin; ns, nonsignificant.

The biochemical properties of the target suggested to us that the target of ATRC-101 might be induced by cellular stress. Multiple types of stress, including oxidative stress, were found to enhance immunoreactivity for ATRC-101 in vitro. EMT6 tumors in mice treated with the chemotherapy doxorubicin exhibited a dramatic dose-dependent increase in ATRC-101 immunoreactivity (*P* < 0.0001; Fig. 5*A*), including at doses of doxorubicin that by themselves had a limited effect on tumor growth. However, evaluation of normal organs showed no appreciable signal following treatment with doxorubicin versus PBS (*SI Appendix*, Fig. S1). The antitumor activity of the combination of doxorubicin with ATRC-101 was also evaluated. Enhanced activity was seen when ATRC-101 and doxorubicin were combined, as evidenced by a significant reduction in tumor growth for ATRC-101 plus doxorubicin compared to PBS, doxorubicin alone, and ATRC-101 alone (Fig. 5*B*).

Cell counting experiments using a semistereological, unbiased approach were used to assess the numbers of immunopositive profiles in tumor cores collected from untreated patients and patients who received anticancer treatment. These experiments demonstrated that anticancer treatment correlated with an increase in the number of ATRC-101–positive malignant cells in colorectal cancer biopsies (Fig. 5 *C*, *Left*). The mean percentages of malignant cells that stained positive for ATRC-101–mIgG2a in the treatment-naïve group and posttreatment group were 49 and 73.5%, respectively. A statistically significant 1.5-fold increase (*P* = 0.027) was observed between the treatment-naïve group and the posttreatment group in the percentage of ATRC-101–mIgG2a-positive malignant cells. However, in breast cancer samples, there was no significant difference between the treatment-naïve group and posttreatment group in the number of ATRC-101-immunoreactive tumor cells (45 vs. 61%, respectively; *P* = 0.125; Fig. 5 *C*, *Right*).

Experiments using archival human formalin-fixed paraffin-embedded tissue samples were used to assess changes in the level of ATRC-101 target, as assessed by an H-score metric, before and after neoadjuvant treatment for breast cancer. Standard-of-care neoadjuvant treatment induced changes in immunoreactivity of ATRC-101 in breast cancer samples (pre- vs. posttreatment; Fig. 5*D*). More than half of the patient samples (56%, 10/18 patients) demonstrated the presence of the target of ATRC-101 at baseline (before treatment). The target of ATRC-101 was also present in over half of the patient samples (56%, 10/18 patients) after treatment. When comparing the matched pre/post sample for each patient, approximately one-third showed no change of the target of ATRC-101 after treatment, one-third showed a decrease, and one-third showed an increase. These data from a small number of highly variable patient samples support a more rigorous investigation of target induction by chemotherapeutics in a controlled trial in humans.

## Discussion

While advances in immunotherapy have drastically changed the treatment landscape in oncology, additional therapies are still needed for those patients whose disease will not respond to available therapies. ATRC-101 is an engineered antibody that targets an extracellular, tumor-specific RNP complex. The discovery of ATRC-101 was dependent on a target-agnostic discovery platform that allows one to reverse the typical target-based drug-discovery paradigm and identify potential tumor-targeting antibodies from a patient demonstrating an ongoing antitumor immune response. The target of ATRC-101 appears to be an RNP complex with PABP-1 as its most prominent component.

The mechanism of action of ATRC-101 suggested by our data are analogous to mechanisms driving immune responses to viral infection and in autoimmune disease (11, 12). Immune activation occurring in response to a virus or the pathogenesis of an autoimmune disease, such as systemic lupus erythematosus, involves TLRs recognizing viral RNA or self-nucleic acids; this may lead to DC maturation, production of immunomodulatory cytokines, DC activation and antigen presentation, and cross-priming of antigens in the major histocompatibility complex class I pathway, followed by generation of adaptive immune responses (11, 12, 20). Similar effects were observed in this preclinical study, as ATRC-101 increased ISG expression in tumors, decreased cDC2 cells, increased cDC1 cells in the blood, drove a shift in macrophages toward the M1 phenotype in tumor, and required CD8^+^ T cells for antitumor activity. In addition, DCs appeared to be activated when their Fc receptors engaged target-bound ATRC-101. It is possible that the RNA associated with the target of ATRC-101 engages one or a combination of TLR-3/7/8 (the TLRs activated by RNA) to promote DC activation, costimulatory receptor expression, cross-priming, and activation of an antitumor CD8^+^ T-cell response. The presence of RNA at the cell surface and potential mechanisms underlying this localization have been described previously (21–25), and future studies will examine the exact nature of this phenomenon for the target of ATRC-101. Of note, TLR3, in particular, can be activated by mRNA (26), which is likely present in the target of ATRC-101 given the abundance of PABP-1 found in the complex. These data and the lack of activity of ATRC-101 seen in T-cell-deficient mice or when mIgG2a replaces mIgG1 support the hypothesis that ATRC-101 stimulates both innate and adaptive immunity to induce effective antitumor responses.

The increase in cDC1 cells with ATRC-101 is consistent with other studies that have reported the role of cDC1 in cancer immunity (27–29). cDC1 cells transport tumor antigens for presentation to naïve CD8^+^ T cells and produce chemokines in the tumor microenvironment that recruit CD8^+^ effector T cells (29). In addition to regulating cytotoxic T-cell recruitment and stimulation, the production of chemokines by cDC1 cells may also result in the recruitment of other cells, such as NK cells, into tumors (29). In addition, cDC1 have been shown to be associated with prolonged overall survival in eight out of 14 solid tumor types (28). ATRC-101 demonstrated antitumor activity alone and in combination with chemotherapy. Preclinical murine studies that evaluated the combination of chemotherapy or immunotherapy with known cDC1 boosters have found a decrease in tumor progression and enhanced cytotoxic T lymphocyte (CTL) activation as well as increased maturation of cDC1 (27). This is consistent with the findings in this study, where a significant reduction in tumor growth was found with the combination of ATRC-101 and doxorubicin compared with either agent alone.

**Fig. 6.**
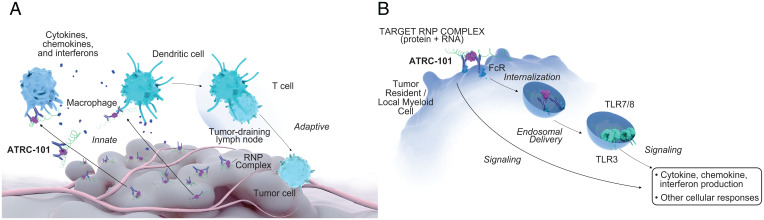
Proposed mechanism of action of ATRC-101. (*A*) Systemically dosed ATRC-101 delivers tumor antigen to cells of the innate immune system, including ATRC-101–target associated RNA that activates DCs and myeloid cells via TLRs, leading to a remodeling of the tumor microenvironment and an adaptive immune response against the tumor. (*B*) The FcR on the tumor resident myeloid cell interacts with target-bound ATRC-101, which leads to endosomal delivery of the ATRC-101 target RNA complex, resulting in TLR signaling that activates DCs and other myeloid cells to express costimulatory molecules and proinflammatory cytokines and to activate antitumor CD8^+^ T-cell responses. FcR, Fc receptor.

The preclinical data reported herein are consistent with a model in which ATRC-101 has antitumor activity by binding to its RNP target on the tumor cell surface and, most immediately, engaging resident myeloid cells in the tumor microenvironment. ATRC-101 interacts with Fc receptors on those myeloid cells while binding its target RNP complex on tumor cells, leading to a release of chemokines/cytokines and likely increased antigen cross-presentation to T cells, finally resulting in an adaptive immune response that likely underlies the immune memory observed (Fig. 6*A*). The engagement of the Fc receptor on the tumor-resident myeloid cells by ATRC-101 bound to its target likely facilitates target RNP internalization, leading to endosomal delivery and, ultimately, TLR signaling (Fig. 6*B*).

These data support a more rigorous investigation of ATRC-101 in a clinical trial and has led to an ongoing phase 1 study (NCT04244552).

## Materials and Methods

See *SI Appendix* for a detailed version of *Materials and Methods*.

### Recombinant ATRC-101 Generation.

The ATRC-101 precursor (i.e., parental; ATRC-101P) was discovered by evaluating tumor-targeting antibodies produced by the plasmablast population of B cells from a patient with stage IV lung adenocarcinoma with stable disease while receiving treatment with anti-PD-1 therapy. Detailed methods for the generation of recombinant ATRC-101 have been previously described (20). The human Fv on mIgG2a antibody for this research study was generated in HEK293 cells.

## Supplementary Material

Supplementary File

## Data Availability

All study data are included in the article and/or supporting information.
