# Supplementary material for: Mobilization of innate and adaptive antitumor immune responses by the RNP-targeting antibody ATRC-101
Source: Proc Natl Acad Sci U S A. 2022 May 4;119(19):e2123483119. doi: 10.1073/pnas.2123483119 (PMC9171637; doi:10.1073/pnas.2123483119)
Supplement: Supplementary File [file pnas.2123483119.sapp.pdf]

PNAS
www.pnas.org

Supplementary Information for

Mobilization of innate and adaptive antitumor immune responses by the RNP-targeting antibody ATRC-101

Alexander Scholz, Jeff DeFalco, Yvonne Leung, Iraz T Aydin, Cathrin J. Czupalla, Wei Cao, Daniel Santos, Nikhil Vad, Shaun M. Lippow, Gilson Baia, Michael Harbell, Judevin Sapugay, Danhui Zhang, Dai-Chen Wu, Erin Wechsler, Anne Z Ye, Jenny W Wu, Xiao Peng, John Vivian, Hargita Kaplan, Rodney Collins, Ngan Nguyen, Mark Whidden, Dongkyoon Kim, Carl Millward, Jonathan Benjamin, Norman M Greenberg, Tito A Serafini, Daniel E Emerling, Lawrence Steinman, William H Robinson, Amy Manning-Bog

Corresponding author:
Lawrence Steinman
Email: steinman@stanford.edu

This PDF file includes:

Supplementary text
Figure S1
SI References

Materials and Methods

Human Tissue Samples

Human tumor, normal adjacent, and normal tissues were obtained as formalin-fixed paraffin-embedded (FFPE) from USBiomax (Derwood, MD), TriStar (Washington, DC), Marin Medical Laboratories (Novato, CA), and Discovery Life Sciences (Huntsville, AL). Fresh frozen samples came from ProteoGenex (Culver City, CA) and BioChain (Newark, CA). All human samples were procured after informed consent and under human subject protocols approved by the respective Institutional Review Board.

Immunofluorescence

Immunofluorescence staining was performed following a standard protocol on 5 µm FFPE tissue sections of commercial human cancer tissues, commercial human normal tissues, or mouse EMT6 tumors. The tissue sections were first dewaxed in xylene and then rehydrated. Antigen retrieval was performed under high pressure at 110°C for 20 minutes using Borg Decloaker buffer (Biocare Medical, Pacheco, CA) in a Decloaking Chamber^{TM} NxGen (Biocare Medical). The sections were treated with blocking buffer (3% bovine serum albumin [BSA] + 3% normal donkey serum in 1X phosphate-buffered saline; PBS) for 1 hour at room temperature. After blocking, the slides were incubated with Alexa Fluor 647-conjugated ATRC-101-mIgG2a or mIgG2a isotype control, at either 5 µg/mL or 10 µg/mL overnight at 4°C. In a subset of experiments, adjacent sections were incubated in anti-polyA binding protein-1 (Abcam) using the manufacturer's recommendations. After rinsing in Wash Buffer (ThermoFisher Scientific, Waltham MA), slides were incubated in donkey anti-rabbit conjugated to Alexa Fluor 488 (1:200) or donkey anti-rabbit conjugated to Alexa Fluor 647 (1:200) for 30 minutes at room temperature. The slides were subsequently washed with Wash Buffer, counterstained with Hoechst, mounted in Pro-Fade aqueous mounting medium (Vector Laboratories, Burlingame, CA), and coverslipped. Images were acquired using an Axio Scan whole slide scanner (Carl Zeiss Microscopy, Oberkochen, Germany).

Fresh frozen tissue sections of hormone receptor-positive breast cancer were sectioned at 8 μm on slides and maintained at –80°C until use, to assess if immunoreactivity for the targets of ATRC-101 were sensitive to RNA degradation. Slides were dried using room temperature air for 5 minutes to minimize endogenous RNase activity and then pre-treated with either PBS, RNase A (0.07 U/mL or 1.4 U/mL), RNase H (1.7 U/mL or 33 U/mL), or DNase I (20 U/mL) for 5 minutes at 37°C. Slides were rinsed with PBS, blocked with 3% normal donkey serum at room temperature for 30 minutes, and incubated in Alexa Fluor 647-conjugated ATRC-101-mIgG2a and anti-E-cadherin (overnight at 4°C). After rinsing in PBS, slides were incubated in donkey anti-rabbit conjugated to Alexa Fluor 488 (1:200) for 30 minutes at room temperature. Slides were counterstained with Hoechst dye, rinsed with PBS, and coverslipped using an aqueous mounting medium. Images were acquired using an Axio Scan whole slide scanner (Carl Zeiss Microscopy).

Immunohistochemistry in Mouse Tissues

Punch biopsies obtained from normal mouse tissues including heart, liver, lung, kidney, brain, spleen, pancreas, and stomach were collected and arranged in a microarray along with tumor tissue and sectioned at 8 μm. The frozen tissue sections were fixed with 4% paraformaldehyde. Signal was detected using the Mouse on Mouse (M.O.M.) ImmPRESS® HRP (Peroxidase) Polymer Kit (Vector Laboratories) and Betazoid DAB Chromogen Kit (BioCare Medical). Briefly, endogenous mouse IgG was blocked with M.O.M. Mouse IgG Blocking Rreagent. Tissue sections were incubated overnight at 4°C with 0.5 μg/mL ATRC-101 or mouse IgG2a isotype control followed by Vector M.O.M. ImmPRESS reagent and Betazoid DAB development. Images were acquired using an AxioScan whole slide scanner (Carl Zeiss Microscopy).

Immunofluorescence Staining for ATRC-101 and Indication-Specific Malignant Cell Markers in Serial Sections of Human Colorectal and Breast Tumors

Colorectal cancer (CRC), non-small cell lung cancer (NSCLC), and breast cancer tissue microarray (TMA) serial sections were stained with ATRC-101-mIgG2a or a malignant cell-

specific cocktail for CRC or breast carcinomas. ATRC-101-mIgG2a immunofluorescence staining was performed on tissue sections of commercial human TMAs following a standard protocol. The primary antibodies were conjugated with the Alexa Fluor 647 as above. The tissue sections were prepared as detailed in the immunofluorescence section previously.

A serial section to the ATRC-101-mIgG2a stained TMA was incubated with an indication-specific malignant cell antibody cocktail. The tissue sections were prepared as previously described. The sections were incubated with Bloxall (Vector Laboratories) for 15 minutes at room temperature, washed with Wash Buffer (Thermo Fisher Scientific) , and incubated in blocking buffer for 1 hour at room temperature. The sections were then incubated with an indication-specific malignant cell cocktail overnight at 4°C. The slides were subsequently washed with wash buffer and incubated with a cocktail of PowerVision Poly-HRP anti-rabbit and anti-mouse secondary antibodies for 30 minutes at room temperature. The slides were then washed with Wash Buffer, and Alexa Fluor™ 647 Tyramide Reagent was applied for 3 minutes. The slides were subsequently washed with wash buffer (Thermo Fisher Scientific), counterstained with Hoechst dye, mounted in PermaFluor Aqueous Mounting Medium (Thermo Fisher Scientific), and coverslipped. Images were acquired using an AxioScan whole slide scanner (Carl Zeiss Microscopy).

Analysis of Tissue Staining

A digital pathology platform (HALO, Indica Labs, Albuquerque, NM) was utilized to perform a semi-quantitative analysis of tumor cell marker and ATRC-101-mIgG2a immunoreactivity.

For serial-stained tumor groups (CRC and breast cancer), the tumor-specific, marker-stained slides were first analyzed by the HALO HighPlex FL algorithm to determine the malignant cells. Then, serial sections stained with ATRC-101-mIgG2a were annotated manually to delineate malignant cells based on a side-by-side comparison of the ATRC-101-mIgG2a and malignant cell marker-stained slides. Subsequently, the HALO HighPlex FL algorithm was applied to determine the total number of malignant cells and the number of ATRC-101-mIgG2a-positive

malignant cells. ATRC-101-mIgG2a positive malignant cell percentage was calculated using the total malignant cell counts and the ATRC-101-mIgG2a-positive malignant cell counts for each sample. *P*-values were calculated using unpaired *t*-test, and tests were considered statistically significant when the $P<0.05$.

Immunohistochemistry (IHC)

IHC staining for ATRC-101 target was performed by an external clinical research organization with expertise in developing, validating, and performing IHC studies (Discovery Life Sciences/Legacy QualTek Molecular Labs, Newtown, PA) using a clinical research antibody with the same human Fv region as the therapeutic antibody on a mIgG2a N297A mutant Fc.

IHC H-score

A board-certified pathologist assessed breast cancer FFPE tissue sections by IHC using standard H-score methodology with a clinical research antibody identifying the target of ATRC-101 to determine changes before and after neoadjuvant treatment. The main components of H-scores are percentages of tumor cells in categories based on the degree of positive staining. Any surrounding staining in the stroma, areas of non-tumor, adjacent normal tissue, and ischemic or necrotic areas are excluded. The percentage of tumor cells with positive staining at a corresponding differential intensity is scored on a four-point semi-quantitative scale (0= null, negative, or nonspecific staining, 1+= low or weak staining, 2+= medium or moderate staining, and 3+= high or strong staining). The percentage of tumor cells staining at each intensity is directly estimated. The H-score was calculated as the sum of the percentage of cells with an intensity of expression multiplied by the corresponding differential intensity; thus, H-scores ranged from 0 to 300.

$$\text{H-Score} = [(\% \text{ at } <1) \times 0] + [(\% \text{ at } 1+) \times 1] + [(\% \text{ at } 2+) \times 2] + [(\% \text{ at } 3+) \times 3]$$

Immunoprecipitation from A549 Whole Cell Extracts and Cross-Linked Extracts

A549 cells were grown according to ATCC specifications, lysed in radioimmunoprecipitation assay (RIPA) buffer, and cleared. Supernatant was incubated at 4°C with ATRC-101P cross-

linked to magnetic Dynabeads™ M-280 Tosylactivated (Invitrogen™) according to manufacturer's recommendation. After incubation, beads were collected on magnets and washed 4 times in PBSN (phosphate-buffered saline [PBS] supplemented with 0.1% NP-40) with sodium chloride added to a final concentration of 0.4 M followed by PBSN washes. Bound antigen was eluted from antibody by incubating in 1% deoxycholate. Samples were evaluated by denaturing SDS-PAGE followed by silver staining and a subset submitted for nanoLC-MS/MS.

A549 lysate was prepared and incubated with ATRC-101P-conjugated M-280 Dynabeads™. Antigen bound to the antibody was crosslinked by adding DTSSP (3,3´-dithiobis(sulfosuccinimidylpropionate)) to the lysate-bead suspension at a final concentration of 1.25 mM and incubating at room temperature for 30 minutes. The reaction was quenched by addition of 40 mM Tris for 15 minutes at room temperature. RNase A was added over a concentration range and incubated for 1 hour at room temperature. Beads were collected on magnets, washed 4 times with PBSN + 0.4 M sodium chloride, then once with PBSN. Bound complexes were eluted with 150 mM ammonium hydroxide and lyophilized by a SpeedVac™ concentrator. Samples were evaluated by denaturing SDS-PAGE followed by silver staining and a subset submitted for nanoLC-MS/MS.

CT26 Mouse Model

. Female, 6-week-old BALB/c mice were inoculated in the right hind flank by subcutaneous injection with 1×10^6 CT26 cells/mouse in 0.2 mL Roswell Park Memorial Institute (RPMI medium without supplements. The day of cell inoculation was designated as Study Day 0. Mice were injected twice weekly for a total of ten doses. For the rechallenge assessment, mice that showed complete regression following ATRC-101P-mIgG2a administration and 20 age-matched naïve control mice were inoculated with 1×10^6 CT26 cells/mouse in 0.2 mL RPMI medium without supplements in the left hind flank by subcutaneous injection on Study Day 99.

EMT6 Mouse Tumor Model

Mouse EMT6 tumor cells were propagated in culture by passaging cells every 2–3 days (1:10 subcultures) for 8 passages. On the day of inoculation, cells were collected, counted, and diluted to 5×10^6 cells/mL in Waymouth's medium without supplements.

Female, 6-week-old BALB/c mice were inoculated in the right hind flank by subcutaneous injection with 1×10^6 EMT6 cells/mouse in 0.2 mL Waymouth's media without supplements. The day of cell inoculation was designated as Study Day 0. Mouse tumors routinely became visible and palpable 3 days after cell inoculation. Tumor volumes were measured twice prior to volume-matched distribution randomization, which occurred on Day 6.

For efficacy studies, mice with established EMT6 tumors were dosed intraperitoneally (IP) with 10 mg/kg ATRC-101-mIgG2a or vehicle (Dulbecco's PBS [DPBS]) on Days 7, 10, 14, 17, and 21 post tumor inoculation with tumor volumes measured a minimum of twice weekly.

For mechanism of action experiments, BALB/c mice with established EMT6 tumors were dosed IP with 10 mg/kg ATRC-101-mIgG2a or vehicle on Days 7, 10, and 13 post tumor inoculation. Blood and tumors were collected from one cohort of mice on Day 7, prior to administration of test article, and cohorts from each treatment group after test article administration on Days 8, 9, 11, 12, 14, or 16 post tumor inoculation.

Mice were monitored for tumor volume, weight, and clinical observations throughout the study.

Animals were euthanized on Days 7 (pre-treatment), 8 and 9 (post dose 1), 11 and 12 (post dose 2), and 14 and 16 (post dose 3) for analysis of the cellular composition of the blood and tissues by flow cytometry, analysis of the tumor microenvironment by immunofluorescence, analysis of cytokine levels by bead-based multiplex assay, or analysis of gene expression by RNA-seq.

Blood and Tissue Harvest for Flow Cytometry and Multiplex Analyses

Mice were anesthetized by isoflurane inhalation, and up to 1 mL of blood was drawn from mice by cardiac puncture using a 25G needle in a 1 mL syringe. Mice were euthanized immediately

after the blood collection by cervical dislocation. A 300 µL aliquot of the blood sample was placed in dipotassium ethylenediaminetetraacetic acid (K2-EDTA) vacutainer tubes (BD Biosciences, Franklin Lakes, NJ), gently inverted up to 10 times and stored at room temperature for up to 1 hour until further processing for analysis by flow cytometry. The remaining blood was aliquoted into a 0.8 mL MiniCollect® serum and plasma tube (Greiner Bio-One, Kremsmünster, Austria). Tubes were left undisturbed in a standing position for 30 minutes. Tubes were spun at 1500 × *g* for 10 minutes to separate the serum. The supernatant was carefully transferred to a 0.5 mL microcentrifuge tube and stored at −80°C until further processing for analysis. Tumors were excised from the mice, weighed, and placed in RPMI media.

Tissue Harvest for Immunofluorescence

Mice were euthanized by carbon dioxide asphyxiation followed by cervical dislocation. Tumors were excised from the mice, weighed, and placed in pre-filled tubes containing 10% neutral buffered formalin (Caplugs). Tumors were fixed for 24 hours at room temperature and then transferred to 80% ethanol prior to embedding.

Tissue Harvest for RNA Sequencing

Prior to euthanasia, all surgical tools and bench surfaces were cleaned with RNaseZap™ (Thermo Fisher Scientific). After processing each mouse, surfaces, gloves, and surgical tools were cleaned with RNaseZap™. Mice were euthanized by carbon dioxide asphyxiation followed by cervical dislocation. Tumors were excised from euthanized mice, weighed, placed in ribonuclease (RNase)-free microcentrifuge tubes, and flash-frozen in liquid nitrogen. Samples were processed for RNA-seq immediately after harvest.

Flow Cytometry Analysis

Blood Processing for Flow Cytometry Analysis

Of the freshly collected blood, 100 µL was blocked with 1 µL TruStain FcX™ PLUS (anti-mouse CD16/32; Biolegend, San Diego, CA) for 10 minutes at 4°C and stained using antibody master mixes for 30 minutes at 4°C with shaking. After staining, 1.6 mL of Lyse/Fix buffer (BD Biosciences) prepared according to the manufacturer's recommendation was added and incubated for 10 minutes at room temperature. Samples were then pelleted by centrifugation at 500 × *g* for 5 minutes, resuspended, and washed with DPBS.

Tumor Dissociation and Processing for Flow Cytometry Analysis

Excised tumors were chopped into smaller pieces and dissociated using a gentleMACS™ Octo Dissociator and mouse Tumor Dissociation Kit per manufacturer's instructions (Miltenyi Biotec, Bergisch Gladbach, Germany). After filtration, cells were aliquoted and frozen in fetal bovine serum (FBS) supplemented with 10% dimethyl sulfoxide. For analysis, cells were gently thawed, washed, and blocked with TruStain FcX™ PLUS (anti-CD16/32; Biolegend). Next, blocked cells were incubated with directly conjugated antibodies for 30 minutes at 4°C while shaking. Cells were washed and counterstained with 1:500 MultiCyt® Membrane Integrity FL1 / BL1 Screening Kit (IntelliCyt, Ann Arbor, MI) before running on a CytoFLEX flow cytometer (Beckman Coulter, Brea, CA). Files were analyzed using FlowJo 10.7.1 software.

Flow Analysis on Human Dissociated Tumor Cells

ATRC-101-mIgG2a was directly conjugated using the SiteClick™ R-PE Antibody Labeling Kit (Thermo Fisher Scientific) and used to stain primary human dissociated tumor cells purchased from Discovery Life Sciences, LLC (Huntsville, AL) In brief, tumor cells were gently thawed, washed, blocked, and incubated with primary-conjugated antibodies. Samples were run on a CytoFLEX flow cytometer (Beckman Coulter) and analyzed using FlowJo 10.7.1 software. Tumor cells from melanoma patients were identified as being $CD45^{-}$. Tumor cells from NSCLC patients were identified as $CD45^{-}$ $CD31^{-}$ $CD90^{-}CD140b^{-}EpCAM^{+}$ E-$Cadherin^{+}$. Tumor cells from Ovarian Cancer were identified as $CD45^{-}CD31^{-}$ $EpCAM^{+}$ E-$Cadherin^{+}$.

Statistical Analyses of Flow Cell Populations

Differences in the means of each cell population between the treatment and control groups were assessed by two-way ANOVA with an interaction term for each post-treatment day relative to pre-treatment baseline at Day 7 using R version 3.4.3 (The R Foundation).

Tumor Processing for Immunofluorescence Analysis

Each fixed tumor was dissected using a slicer matrix with 2.0 mm section slide intervals (Zivic Instruments, Pittsburgh, PA) to assess tumor microenvironment at 3 to 6 evenly spaced locations to account for tumor heterogeneity. The resultant tissue slices from each tumor were placed in tissue cassettes (up to three tumors in one cassette; including a piece of mouse spleen as a landmark for orientation) and subsequently processed in graded alcohols and xylene and embedded in paraffin per manufacturer's instructions using a Tissue Tek VIP® 6 AI Vacuum Infiltration Processor (Sakura Finetek, Torrance, CA) and Tissue-Tek® TEC™ 5 embedding console system (Sakura Finetek). Blocks containing tumors were sectioned at 5 µm using an Accu-Cut® SRM™ 200 Rotary Microtome (Sakura Finetek). Sections were mounted to slide and dried.

Immunofluorescence Staining in Mouse Tumor

Immunofluorescence staining was performed on 5 µm formalin-fixed, paraffin-embedded tissue sections of mouse EMT6 tumors, following standard immunostaining protocols. The primary antibodies were utilized with species-specific secondary antibodies and detected using standard tyramide signaling amplification methodology or biotin-streptavidin signal amplification strategy to stain for T- macrophage populations.

Macrophage Marker Protocol

The tissue sections were dewaxed in xylene and rehydrated. Antigen retrieval was performed under high pressure at 95°C for 20 minutes using Target Retrieval Solution (Dako, Glostrup,

Denmark) in a Decloaking Chamber™ NxGen (Biocare Medical). The sections were blocked for 15 minutes at room temperature with Bloxall (Vector Laboratories). Subsequently, tissue sections were successively blocked for 15 minutes with Avidin block (Biocare), Biotin block (Biocare), and blocking buffer (3% BSA with 3% normal donkey serum in 1X PBS) for 1 hour at room temperature. After blocking, the slides were incubated with primary antibodies against F4/80 and inducible nitric oxide synthase, F4/80 and Arginase-1 (Arg-1), or species-appropriate isotype controls for 1 hour at room temperature. The next day, slides were washed with Wash Buffer (Thermo Fisher Scientific), incubated for 30 minutes at room temperature with species-specific secondary antibody conjugated to biotin, and then 5 minutes at room temperature with Streptavidin Conjugate CF®647 Working Solution (Biotium). The slides were subsequently washed twice with Wash Buffer, incubated with species-specific secondary PowerVision Poly-HRP for 30 minutes at room temperature, followed by incubation with CF Tyramide 488 Working Solution (Biotium) for 3 minutes at room temperature.

All tissue slides were counterstained with Hoechst dye, mounted in PermaFluor Aqueous Mounting Medium (Thermo Fisher Scientific), and coverslipped. Images were acquired using an AxioScan whole slide scanner (Carl Zeiss Microscopy).

Algorithm-based Digital Image Analysis of Mouse Tumor

Positive immunoreactivity was assessed by analyzing whole slide images (Carl Zeiss Microscopy AxioScan) using Indica Labs HALO 2.1 software through the application of the HighPlex FL v3.0.2 or v3.1.0 algorithm to identify the double-positive cells. For each of the 3–6 tumor tissue sections per tumor, regions of interest were manually annotated, while peripheral tissues such as skin, adipose, and connective tissues, as well as tissue folds and other artifacts, were excluded. Individual cells were detected based on Hoechst nuclear stain. For each experiment, the detection thresholds for fluorescent signals were adjusted based on antibody-specific signal strength and nonspecific background signal. Cell count estimates were determined for each tumor and normalized to cell counts observed in the corresponding IgG

antibody-stained tumor. The M1/M2 was assessed by comparison of the cell estimation for each population as determined by the aforementioned algorithm.

Cytokine Analysis by Bead-based Multiplex Assay

According to the manufacturer's instruction, cytokine concentrations in serum samples were analyzed using a custom QBead kit (Miltenyi Biotec) designed to detect 23 cytokines simultaneously. In brief, serum samples were incubated for 1 hour with detection beads followed by1 hour incubation with a detection reagent. After washing, the fluorescent intensity of each bead population was analyzed using an iQue screener plus with ForeCyt software. Standard curves were used to calculate the absolute concentrations of each analyte.

Ribonucleic Acid Extraction from Whole Tumors

Before RNA-seq sample preparation from tumors, RNaseZAP™ (Thomas Scientific) was used to clean bench surfaces and external surfaces of all reagents and tools used for tumor processing, including gloves, pipettes, pipette tip boxes, etc.

Tumor homogenization was performed at room temperature in 1 mL TRIzol™ reagent (Thermo Fisher Scientific) per 50 mg tissue, using a PolyTron Kinematica PT 1200 E hand disperser with a 7 mm Easy Care generator (Thomas Scientific, Swedesboro, NJ). After each tumor, the generator was cleaned with Milli-Q water and 80% ethanol. Homogenates were incubated at room temperature for 5 minutes to allow for the dissociation of nucleoprotein complexes.

RNA was isolated from each homogenate following the manufacturer's protocol for TRIzol™ reagent. All reagents were handled using glass pipets or solvent-safe pipette tips. For each sample, 0.2 mL of chloroform were added per 1 mL of TRIzol™ used for homogenization. The samples were mixed and incubated at room temperature for 3 minutes, then centrifuged at 12,000 × *g* and 4°C for 15 minutes to separate out an RNA-containing aqueous phase. This was transferred to a new tube, and from this point onwards, samples were kept at 4°C to

minimize RNA degradation. To precipitate the RNA, 0.5 mL of isopropanol per 1 mL of TRIzol™ used for tumor homogenization was added to the aqueous phase.

The samples were mixed and incubated at 4°C for 10 minutes and then transferred on dry ice to BGI Genomics (Seattle, WA), which completed the final steps of RNA purification. The RNA was pelleted by centrifugation at 12,000 × *g* and 4°C for 10 minutes and then washed by resuspending in 1 mL of 75% ethanol per 1 mL of TRIzol™ used for tumor homogenization. The RNA was pelleted again by centrifugation at 7,500 × *g* and 4°C for 5 minutes, air-dried for 5–10 minutes, and finally resuspended in RNase-free 0.1 mM EDTA for quality control and sequencing.

Sequencing

BGI Genomics performed quality control and sequencing of the RNA samples. Briefly, using an Agilent 2100 Bioanalyzer and the RNA 6000 Nano Kit, the total RNA samples were analyzed for RNA concentration, 28S/18S RIN (RNA integrity number), and fragment length distribution. Oligo(dT) selection or ribosomal RNA depletion was used to enrich the messenger RNA (mRNA) from each total RNA sample to generate complementary deoxyribonucleic acid (cDNA) libraries for sequencing. The mRNA was fragmented and reverse-transcribed to double-stranded cDNA using random N6 primers. The cDNA was then end-repaired and adenylated at the 3' ends, and bubble adapters were ligated to each end. The cDNA was amplified by a polymerase chain reaction and then denatured by heat. The resulting single-stranded cDNA molecules were cyclized using splint oligos before being sequenced on the DNBSeq™ platform. BGI Genomics transmitted the raw sequencing data to Atreca through deposition into an AWS S3 bucket.

Raw Sequence Data Processing

The raw sequencing data were processed. Quality control (QC) was generated per FASTQ file using FastQC (https://www.bioinformatics.babraham.ac.uk/projects/fastqc/) and harmonized into

a single report with MultiQC (1). Trim Galore! (https://www.bioinformatics.babraham.ac.uk/projects/trim_galore/)/Cutadapt (2) was used to preprocess the reads, and Salmon (3) calculated the gene- and transcript-level quantification results. The MM10 (GRCm38.p6) version of the mouse reference genome (https://www.ncbi.nlm.nih.gov/assembly/GCF_000001635.26) and Gencode M24 gene annotations (https://www.gencodegenes.org/mouse/) were used to generate the inputs to Salmon. FastQC/MultiQC were used to generate the distribution of Phred quality score by read and base position for all samples. Principal component analysis was used to separate samples based on their expression of 48 genes involved in toll-like receptor (TLR) signaling. The samples were plotted as a function of the first and second principal components, PCA1 and PCA2. Gene quantification counts from Salmon were used as inputs to DESeq2 (4) to calculate differential expression values, such as log2 fold change and *P*-values, for all pairwise conditions and timepoints The log2 fold changes between ATRC-101-mIgG2a and the vehicle-treated tumors were compared on Day 9 and Day 12 for TLR-related genes and core vertebrate interferon-stimulated genes (5)

T-Cell Dependence Studies

Antitumor efficacy of ATRC-101P-mIgG2a was assessed in the presence or absence of an anti-mouse CD8α antibody, which had been shown to deplete mouse $CD8^+$ T cells. To assess antitumor efficacy, BALB/c mice were inoculated subcutaneously with EMT6 cells. Once tumors were established, mice were dosed twice-weekly with (i) the T-cell depleting anti-mouse CD8α antibody or vehicle (DPBS) and (ii) ATRC-101P-mIgG2a or vehicle control (DPBS). Mice were monitored for 27 days from inoculation with tumor cells to assess tumor volumes. Antitumor efficacy of ATRC-101P-mIgG2a was also assessed in EMT6 tumor-bearing BALB/c nude mice (BALB/$c^{nu/nu}$ (CAnN.Cg-*Foxn1*^{nu}/Crl)), which lack T cells. BALB/c mice were inoculated subcutaneously with EMT6 cells. Starting 7 days post tumor inoculation, mice were dosed twice weekly with (i) 10 mg/kg ATRC-101P-mIgG2a, (ii) 20 mg/kg of ATRC-101P-mIgG2a, or (iii)

vehicle control (DPBS). Mice were monitored for 25 days from initiation of dosing to assess tumor volumes and survival. This study was performed at Charles River Laboratories (Morrisville, NC).

Fc-effector Function on Antitumor Efficacy of Chimeric Variant

BALB/c mice were inoculated with 1×10^6 EMT6 tumor cells and randomized to treatment groups 6 days later. Starting 7 days post EMT6 inoculation, with a group average mean tumor volume of 94.5 mm^3, mice were dosed IP twice weekly with 10 mg/kg of ATRC-101P-mIgG2a (lot number AB-000196-19), ATRC-101P-mIgG1 (lot number AB000196-17), a chimeric variant of the parental antibody ATRC-101P or vehicle (PBS). The tumor volumes of individual animals from each treatment group are shown (20 animals per group).

Doxorubicin

To evaluate impact of doxorubicin on ATRC-101 reactivity in mouse tumors, BALB/c mice were inoculated SC in the right hind with cultured cells of the EMT6 mouse breast cancer cell line described above to generate the EMT6 syngeneic mouse tumor model. Animals were administered saline or doxorubicin at 2, 5, or 10 mg/kg intravenously (IV) once per week for 3 consecutive weeks and euthanized when maximum tumor burden was reached. Tumors were FFPE, thin sectioned, and used to assess ATRC-101 immunoreactivity using a primary conjugated to AF647 as described in previous sections.

To assess the effects of doxorubicin on the target of ATRC-101 in normal mouse tissues, EMT6 tumor-bearing and non-tumor-bearing BALB/c mice were dosed IP with vehicle (saline) or doxorubicin (2 mg/kg or 10 mg/kg) on Day 1 and Day 8. Normal tissues from the heart, liver, lung, kidney, brain, spleen, pancreas, and stomach were collected 24 hours and 2 weeks after the last dose and snap frozen until use.

Doxorubicin – ATRC-101 Combination

BALB/c mice were inoculated SC in the right hind with cultured cells of the EMT6 mouse breast cancer cell line as described above to generate the EMT6 syngeneic mouse tumor model.

The EMT6 syngeneic mouse tumor model was used to evaluate the impact of administration of ATRC-101-mIgG2a at 3 dose levels (1, 3, or 5 mg/kg) twice weekly IP on tumor volume and

survival when administered alone or in combination with doxorubicin dosed IV at 2 dose levels (2 or 5 mg/kg) once weekly. The impact on mortality, clinical observations, and body weights were also evaluated. Test article administration was initiated 6 days post tumor inoculation by twice-weekly IP injection for a total of 7 doses. Doxorubicin was given once a week by IV injection starting on Day 6 for a total of 3 doses. Test articles were dosed at 10 mL/kg based on individual mouse body weight. Mice not dosed with ATRC-101-mIgG2a or doxorubicin were dosed at 10 mL/kg with saline and PBS following the dosing schedules of both the ATRC-101-mIgG2a and doxorubicin. Tumor volumes were measured using electronic calipers connected to the StudyLog. Tumor length and width were recorded using the 'tumor volume assessment' function of StudyLog, and tumor volumes were calculated automatically using the following equation:

$$\text{Tumor Volume (mm}^3\text{)} = \text{length} \times \text{width}^2 \times 0.5.$$

Throughout the study, all mice were dosed as scheduled or until they were removed from the study based on the euthanasia criteria to assure humane conditions and compliance with established study end points according to animal care and use protocol number EB17-010-104 at Explora Biolabs (South San Francisco, CA).

Statistical Analyses of *In Vivo* Experiments

Survival benefit was assessed by a one-sided log-rank (Mantel-Cox) test using R version 3.4.3 (The R Foundation). Analyses of tumor volumes prior to endpoint were assessed by calculation of the normalized area above the curve (NAAC) and the normalized growth rate metric (NGRM) for each animal. To determine the NAAC, the area between the tumor volume curve and the tumor volume endpoint of 2000 mm^3 was divided by the total area between the first time point at which all animals have a measurable tumor volume and the time point of interest, yielding a value between 0 and 1. To determine the NGRM, the slope of the log-transformed tumor volumes versus time was calculated and then normalized to a value between 0 and 1. Statistically significant differences in distributions of NAAC and NGRM between groups were evaluated with a one-sided Wilcoxon rank-sum test using R version 3.4.3 (The R Foundation).

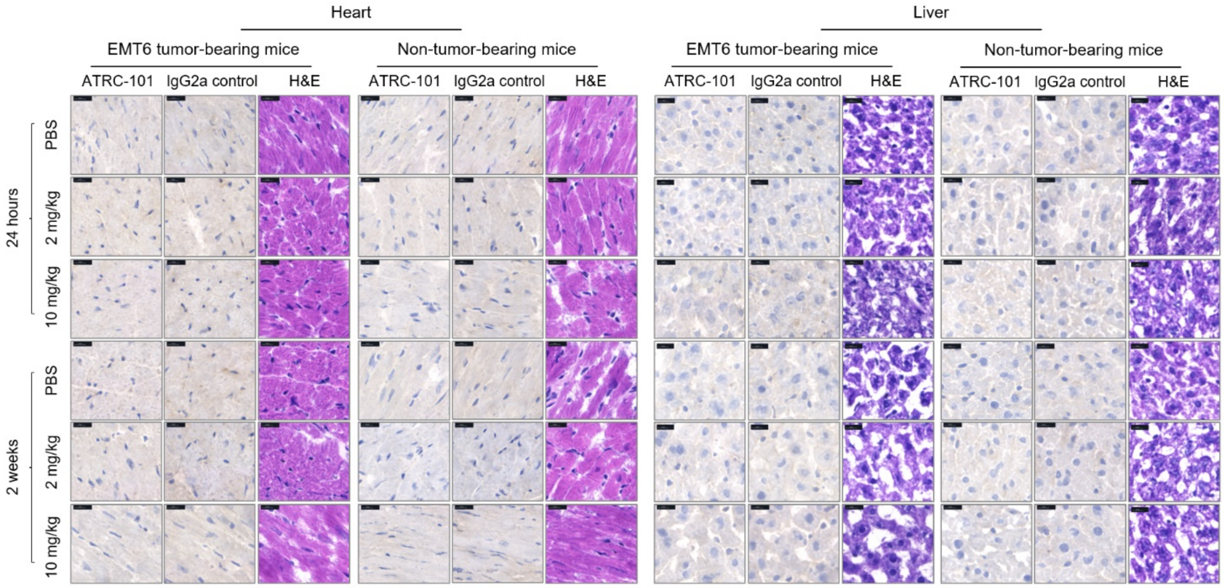

Fig. S1. Impact of doxorubicin on ATRC-101 signal in normal mouse tissues. No appreciable ATRC-101 immunoreactivity was observed in mouse heart and liver after doxorubicin treatment in both EMT6-tumor and non-tumor bearing mice; Bar = 10 μm

IgG, immunoglobulin G; H&E, hematoxylin and eosin; PBS, phosphate-buffered saline.

References SI Appendix

1. P. Ewels, M. Magnusson, S. Lundin, M. Käller, MultiQC: summarize analysis results for multiple tools and samples in a single report. *Bioinformatics* **32**, 3047–3048 (2016).
2. M. Martin, Cutadapt removes adapter sequences from high-throughput sequencing reads. *EMBnet.journal* **17**, 10–12 (2011).
3. R. Patro, G. Duggal, M. I. Love, R. A. Irizarry, C. Kingsford, Salmon: fast and bias-aware quantification of transcript expression using dual-phase inference. *Nat. Methods* **14**, 417–419 (2017).
4. M. I. Love, W. Huber, S. Anders, Moderated estimation of fold change and dispersion for RNA-seq data with DESeq2. *Genome Biol.* **15**, 550 (2014).
5. A. E. Shaw, *et al.*, Fundamental properties of the mammalian innate immune system revealed by multispecies comparison of type I interferon responses. *PLoS Biol.* **15**, e2004086 (2017).
